# HCV and tumor-initiating stem-like cells

**DOI:** 10.3389/fphys.2022.903302

**Published:** 2022-09-15

**Authors:** Keigo Machida

**Affiliations:** Department of Molecular Microbiology and Immunology, Southern California Research Center for ALPD and Cirrhosis, University of Southern California Keck School of Medicine, Los Angeles, CA, United States

**Keywords:** cancer stem cell, tumor-initiating stem-like cells (TICs), drug resistance, HCV, hepatocellular carcinoma (HCC), obesity

## Abstract

Neoplasms contain tumor-initiating stem-like cells (TICs) that are characterized by increased drug resistance. The incidence of many cancer types have trended downward except for few cancer types, including hepatocellular carcinoma (HCC). Therefore mechanism of HCC development and therapy resistance needs to be understood. These multiple hits by hepatitis C virus (HCV) eventually promotes transformation and TIC genesis, leading to HCC development. This review article describes links between HCV-associated HCC and TICs. This review discusses 1) how HCV promotes genesis of TICs and HCC development; 2) how this process avails itself as a novel therapeutic target for HCC treatment; and 3) ten hall marks of TIC oncogenesis and HCC development as targets for novel therapeutic modalities.

## 1 Introduction

### 1.1 Hepatitis virus infection is a major risk factor of hepatocellular carcinoma

Hepatitis B and C virus (HBV/HCV) infection, alcoholism and obesity are major risk factors for hepatocellular carcinoma (HCC) (Okuda, 2000; Crippin et al., 2002). Among all the risk factors, HCV infection is a major and highest risk factor for developing HCC because it promotes fibrosis and cirrhosis (El-Serag and Rudolph, 2007). Approximately 90% of HCV-associated cancers present in advanced fibrosis or cirrhosis. Other nonviral factors (such as alcoholism and obesity) account for about 20% of HCC cases (El-Serag and Mason, 1999) since diabetes and obesity are the strongest metabolic factors associated with HCC (Hagstrom et al., 2018). The incidence of liver cancer is rising with an estimated 841,080 (4.7%) new cases and 781,631 deaths for 2018 (Bray et al., 2018; Gerbes et al., 2018; Kulik et al., 2018; Roche et al., 2018). HCV infection is present in around 50% of cases and the incidence of HCV-induced HCC is falling (Roche et al., 2018).

Treatment options for HCC are limited and not encouraging. The 3-years survival rate of HCC is 13%–21% without any curative treatment (Ebara et al., 1986; Barbara et al., 1992). The 5-years survival rate of HCC is less than 5% with or without therapeutic intervention (El-Serag and Mason, 1999), even in advanced countries such as the United States. (Okuda, 2000; Liang and Heller, 2004). Since the incidence rate of extrahepatic metastasis is 13% at 5 years (Kanda et al., 2008), liver resection is a viable option for HCC combined with cirrhosis (Nakamura et al., 2014). However, only 10%–23% of HCC patients are candidates for surgery (Sonnenday et al., 2007; Shah et al., 2011). Thus, HCV-associated HCC remains an incurable malignancy and an urgent unmet medical need. TIC-mediated HCC development are clinically important. The lifetime risk of HCC in chronically infected HCV individuals is 2%–7% (Di Bisceglie et al., 2003), although it may take 30–40 years for HCC to develop in these patients. Furthermore, HCV affects more than 75 million people worldwide (Okuda, 2000; Yao and Terrault, 2001; Okuda et al., 2002). The main risk factors for developing HCC are viral hepatitis infections such as HBV and HCV. However, the incidence of HCC is rising in non-alcoholic fatty liver disease (Holmes and Chung, 2016; Suresh et al., 2020; Huang et al., 2021).

### 1.2 Chronic liver damage caused by viral infection and environmental factors

**FIGURE 1:**
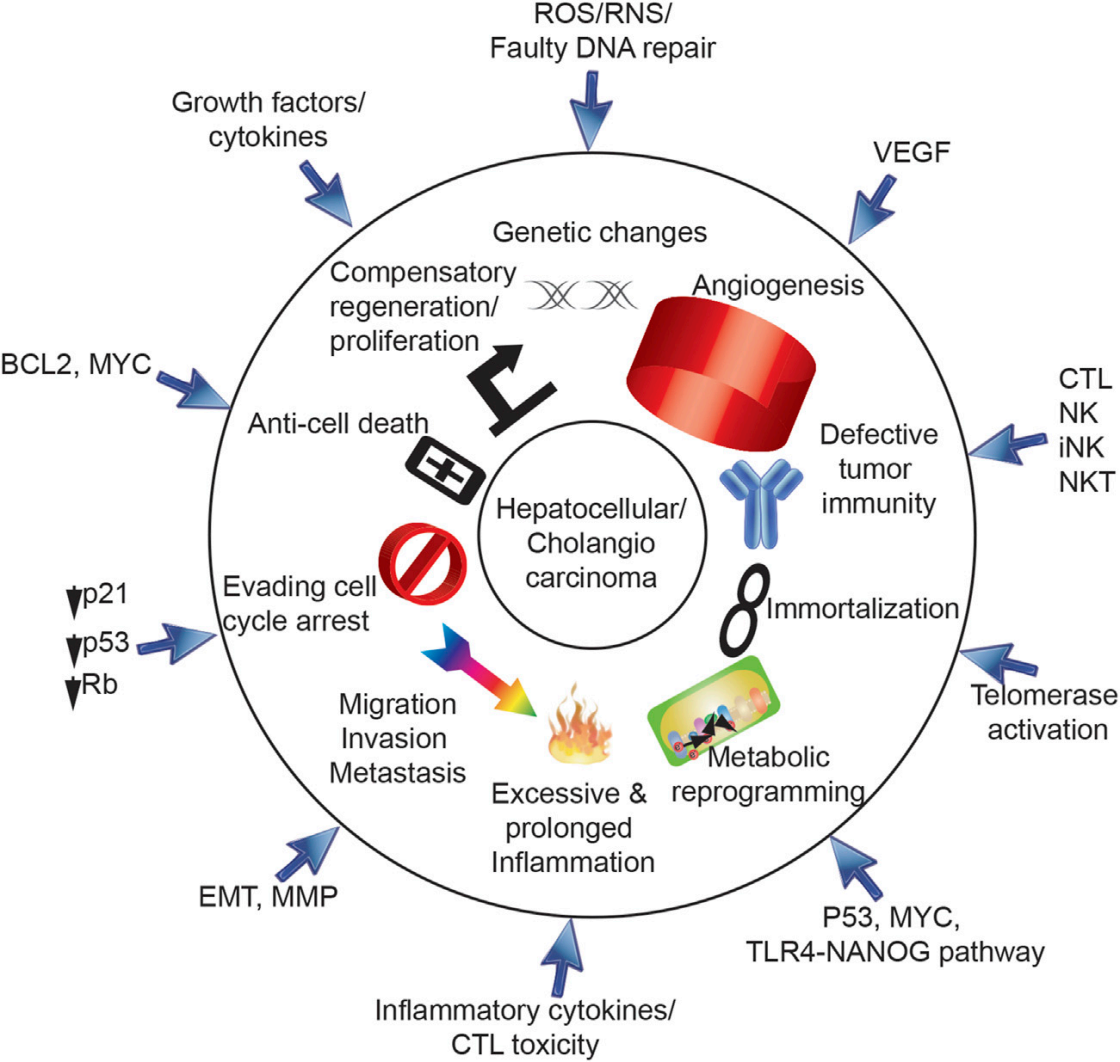
Ten hallmarks for cancer development via HCV. Ten hallmarks of cancer are triggered by HCV, including 1) genetic changes, 2) angiogenesis, 3) defective tumor immunity, 4) immortalization, 5) metabolic reprogramming, 6) excessive and prolonged inflammation, 7) migration/invasion/metastasis, 8) evading cell cycle arrest, 9) anti-cell death and 10) compensatory regeneration/proliferation. HCV core protein, E1, E2, NS3, NS5A, and NS5B are involved in progression of HCV-induced HCC.

Chronic liver damage caused by viral infection and environmental factors (such as alcohol or metabolic syndrome) can result in increased risk for HCC. The cirrhotic liver is a permissive factor for HCC due to the large regenerative activity repetitive damage-regeneration cycles or formation of dysplastic nodules (International Consensus Group for Hepatocellular Neoplasia, 2009). Thus, the cirrhotic liver may be considered a pre-neoplastic “cancer field” comprised of genetically abnormal but non-neoplastic tissue that is at high risk for malignant transformation (Figure 1) (Braakhuis et al., 2003). Understanding the molecular mechanisms of HCV-induced hepatocarcinogenesis will aid in the development of improved therapeutic modalities (Crippin et al., 2002).

HCV-associated cirrhosis can result in HCC by ultimately promoting tumor-initiating stem-like cells (TIC) formation since deposition of extracellular matrix (ECM), including collagen and laminin, promotes tumor-prone microenvironment. These TICs can develop into several different types of liver cancer, e.g., HCC and cholangiocarcinoma (CC). TICs are resistant to conventional chemotherapy and immunotherapy and persist as recurrent tumors or circulating tumor cells. TICs share key features with embryonic stem cells (ESCs) present in preimplantation blastocyst stage embryos, including the expression of a core pluripotency-associated transcription factor (TF) network (Kim et al., 2010; Ikushima et al., 2011). Liver progenitor cells have asymmetric cell division process. In untransformed stem cells, self-renewal occurs through asymmetric cell division, in which one daughter cell retains the multipotent progenitor status of its parent while the other cell commits to a specialized cell fate. In contrast to ESCs, TICs fail to control the self-renewing mode of cell division. As asymmetric cell division mechanism is disrupted in HCCs, TICs exhibit a loss of this intrinsic asymmetry without regulated differentiated daughter cells, leading to the ectopic implementation of stem cell gene expression programs in both progeny cells. This leads to subsequent unchecked expansion of the progenitor cell pool (Cicalese et al., 2009; Knoblich, 2010; Martin-Belmonte and Perez-Moreno, 2012). Thus, understanding the mechanism of genesis of TICs paves the way for novel therapeutic approaches, including cell fate-determinant molecule NUMB, which is p53-MDM2 associated proteins. This key cell fate determinant molecules are targeted by interacting protein TBC1D15 in TICs (Feldman et al., 2013).

### 1.3 Ten hallmarks of cancer are triggered by HCV in hepatocytes

**FIGURE 2:**
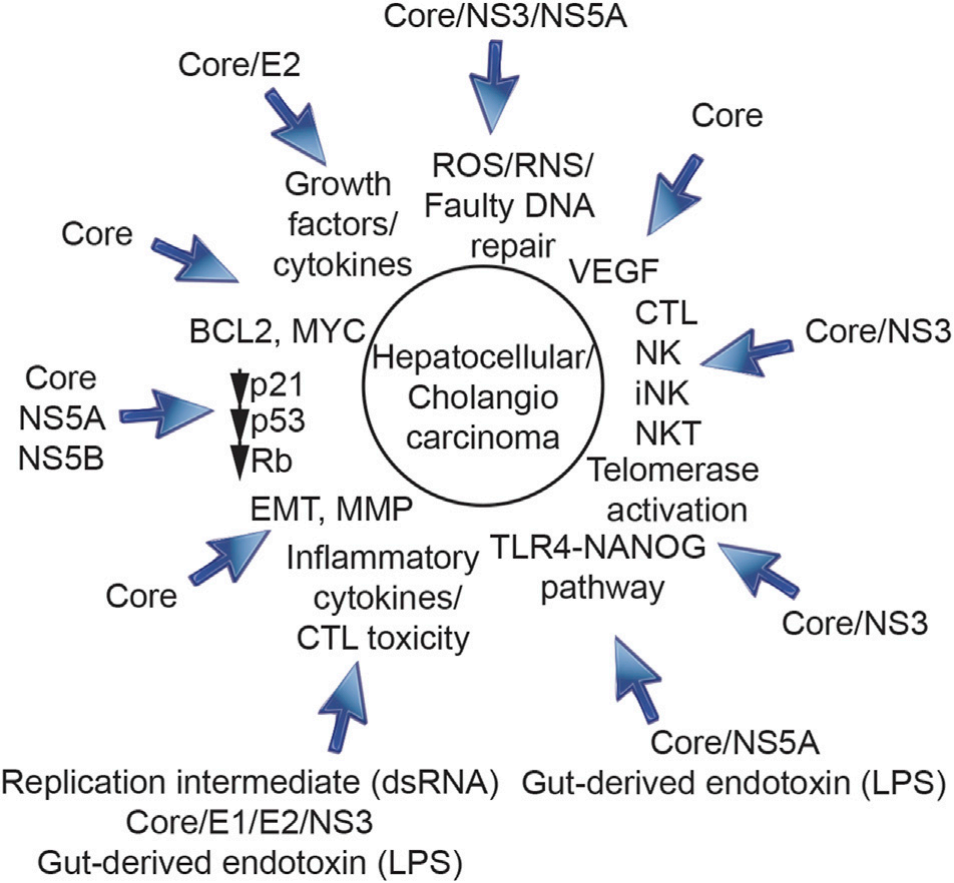
Roles of HCV structural proteins (core protein, E1, E2) and non-structural proteins (NS3, NS5A, and NS5B) in progression of HCV-induced HCC. HCV core protein, E1, E2, NS3, NS5A, and NS5B are involved in initiation, promotion and progression of HCV-induced HCC and genesis of TICs.

Ten hallmarks of cancer (Hanahan and Weinberg, 2011) are triggered by HCV in hepatocytes (Figures 1, 2), including 1) genetic changes, 2) angiogenesis, 3) defective tumor immunity, 4) immortalization, 5) metabolic reprogramming, 6) excessive and prolonged inflammation, 7) migration/invasion/metastasis, 8) evasion of cell cycle arrest, 9) anti-cell death and 10) compensatory regeneration/proliferation. Following ten sections describe ten hall marks of genesis of TICs induced by HCV infection and/or comorbidity, including environmental factors, such as alcoholism, obesity, cirrhosis and xenobiotic agents.

The following text enumerates HCV connections to a series of cellular “Cancer Hallmarsks.” This is important since multiple hits transforms hepatocytes to TIC and HCC. Understanding links between cancer hallmarks and TIC genesis makes conceptual advances to help advance cancer research and therapy by clarifying novel targeting therapy and innovative strategy to suppress theHCC recurrence problems and metastatic spread of HCC cells.

## 2 Genetic changes

### 2.1 Chromosome translocations

Defects in DNA repair genes cause genetic instability, gross chromosomal rearrangements and accumulation of mutations, leading ultimately to neoplastic transformation. Both homologous recombination and nonhomologous end joining (NHEJ) play a role in the repair of double-strand DNA breaks (DSBs) in mammalian cells (Hiom, 1999). The interaction of broken DNA with members of the Rad52 epistasis group, including Rad51, a mammalian homologue of bacterial RecA, initiates homologous recombination repair (Hiom, 1999). Following DNA damage, Rad51 is redistributed within the nucleus (Haaf et al., 1995; Baumann and West, 1998) and induces the ATP-dependent homologous strand pairing reaction that initiates recombination. In contrast, NHEJ works by non-homology-dependent ligation of broken DNA ends. DNA-dependent protein kinase (DNA-PK) and its associated proteins Ku70, Ku80, and Xrcc4 mediate NHEJ (Song et al., 2003).

Structural variations (STVs) of chromosomes include translocation, deletion, or inversion of chromosomes of gene *APC*, and tandem duplications (Fujimoto et al., 2016). HCCs contain broad genomic gains (1q, 5p, 6p, 8q, 17q, 20q, and Xq) and deletions (1p, 4p-q, 6q, 8p, 13p-q, 16p-q, 17p, 21p-q, and 22q) (Totoki et al., 2011; Guichard et al., 2012; Kan et al., 2013; Ahn et al., 2014; Schulze et al., 2015), suggesting that STVs increase the expression of oncogenes and/or decrease the expression of tumor suppressor genes to promote hepatocarcinogenesis.

The take-away message is that HCV-associated HCCs have frequent chromosomal aberrations. These frequent chromosomal aberrations can be targeted and translated into therapies. The status of current related therapeutic strategies is under development. HCV-associated HCCs have frequent MYC loci amplification. These constitutive MYC activation can be targeted and translated into therapies.

### 2.2 Variations in *TP53, CTNNB1, ARID1A,* and non-coding regions

Mutations in tumor-suppressor genes or proto-oncogenes or the activity of growth factors during chronic HCV infection transforms hepatocytes, cholangiocytes, and liver progenitor cells (Simonetti et al., 1992). Whole genome and exome analysis demonstrated that *TP53*, *CTNNB1,* and chromatin modulators, including *ARID1A* and *ARID2*, are the most frequently mutated coding genes in HCC (Shibata and Aburatani, 2014; Totoki et al., 2014; Fujimoto et al., 2016). Loss-of-function *ARID1A* mutations are correlated with poor prognosis, sorafenib resistance, HCC invasion and metastasis (Nhieu et al., 1999). The most frequently mutated driver genes in human alcohol-associated HCCs, but not in dysplastic macronodules (Guichard et al., 2012), are in the chromatin remodeling complex (*ARID1A*) (8%–38%), β-Catenin/Wnt (activating mutations in exon 3 of *CTNNB1*), and *TP53* (30%–65%). *CTNNB1* point mutations occurred in serine residues (Ser 33, 37, and 45) and through the destruction complex (GSK3β and CSNK1A1). The more frequent mutations in non-coding regions are found in the *TERT* promoter and *TFPI2*. Long intergenic noncoding RNAs, including *NEAT1* and *MALAT1*, are also frequently mutated (Fujimoto et al., 2016). Genomic gain of function causes focal amplifications in cancer-related genes such as *VEGFA* and *FGF3/4/19/CCND1*, which is associated with a good response to the multi-kinase inhibitor sorafenib (Arao et al., 2013; Llovet, 2014). Point mutations and also STV breakpoints in HCC tissues are detected in cancer-driver genes, including *TERT*, *ARID1A*, *ARID2*, and *PTEN*, (Fujimoto et al., 2016). Therefore, both nucleotide variants and STVs of chromosomes are detected in hepatitis virus-related HCCs. These diver mutations direct self0renewal ability of TICs.

The take-away message is that tumor driver gene mutations in *TP53, CTNNB1, ARID1A* make hepatocytes susceptible for HCC development. These driver mutation are targeted and translated into therapies, including ICG-001 for CTNNB1 mutation (Delgado et al., 2014; Lin et al., 2016) and Adenovirus expressing functional p53 (Anderson et al., 1998). The status of current related therapeutic strategies showed promising responses for ICG-001 therapy and Ad-p53 (Anderson et al., 1998).

### 2.3 Induction of mutator phenotype

HCV-associated HCC has a 3–5 times higher mutation rate than associated non-tumor liver tissues. Furthermore, the ratio of amino acid replacement and silent mutations in the tumors, but not in the neighboring non-tumor tissues, was significantly higher than the ratio expected in the absence of selection of growth advantage of premalignant cells (Machida et al., 2004a) since chronic hepatocellular turnover could select for cells with genetic or epigenetic changes that confer growth advantages allowing clonal expansion. For example, HCV infection induced a mutator phenotype, which involves enhanced mutations in many somatic genes, including immunoglobulin (Ig) genes, proto-oncogenes and tumor suppressor genes (Machida et al., 2004a). HCV infection promotes error-prone DNA polymerase expression and increases mutation frequencies by induction of ROS and reactive nitrogen species and by inhibition of DNA repair mechanisms (Machida et al., 2004a). Therefore, HCV-induced mutations contribute to the eventual selection and amplification of certain deleterious mutations of proto-oncogenes or tumor suppressor genes in tumors. Accordingly HCV-associated oncogenesis is characterized by a long latency period due to the need for multiple hits.

These mutator phenotype can be great target for immune checkpoint inhibitor since high genetic instability is good prognosis maker for immune checkpoint inhibitor. Indeed, immune checkpoint inhibitor Anti-PD-1 therapy got FDA approval after landmark clinical trial (El-Khoueiry et al., 2017).

### 2.4 Reactive oxygen species

Viral or immune-mediated reactive oxygen species (ROS) induce oxidative stress. ROS-associated oxidative DNA damage (such as 8-oxo-dG) promotes DNA mutagenesis, leading to oncogenic transformation in chronic hepatitis C (Figure 2). For example, the HCV core protein promotes formation of intracellular ROS, both *in vitro* (Okuda et al., 2002) and *in vivo* (Korenaga et al., 2005), via its localization to the mitochondria and inhibition of electron transport (Korenaga et al., 2005). The electron flow that interacts with oxygen molecules results in formation ROS prior to reaching the cytochrome oxidase complex. HCV infection induces ROS (Choi and Ou, 2006), which leads to oxidative DNA damages and lipid peroxidation in HCV-infected cells (Machida et al., 2006). Consequently pretreatment of HCV-infected cells with ROS inhibitors prevented mitochondrial damage and the production of ROS (Machida et al., 2006). ROS with NO (Machida et al., 2004b) induces steatosis (through lipid peroxides) and oncogenesis (through DNA mutations and STAT3 activation), leading to acute hepatocyte damage (*via* production of STAT3) (Machida et al., 2006). Individual liver cells undergo disease evolution to reproduce HCV-associated HCC (Salk et al., 2010). Activated inflammatory cells (CTL or NK) also release ROS and nitrogen (RNS) species and induce lipid peroxidation (Bartsch and Nair, 2006), leading to a pro-carcinogenic microenvironment. Viral particle was consisted with structural proteins, nucleocapside Core, Envelop genes E1 and E2 and p7. Non-structural proteins are consisted of Serine protease NS2 and NS3, protease cofactor NS4A, NS4B, NS5A, and RNA-dependent RNA polymerase NS5B (Holmes and Chung, 2016). The expression of some HCV proteins, in particular structural nucleocapside protein core and non-structural viral protein NS5A, may also contribute directly to the induction of oxidative stress (Figure 2).

The take-away message is that HCV-associated ROS production promotes DNA damages and inhibits DNA repair, leading to mutator phenotypes. These HCV-associated mutaor phenotype can be targeted by vitamin treatment. However current clinical reports are not optimistic for HCC patients.

### 2.5 Inhibition of DNA damage repair

**FIGURE 3:**
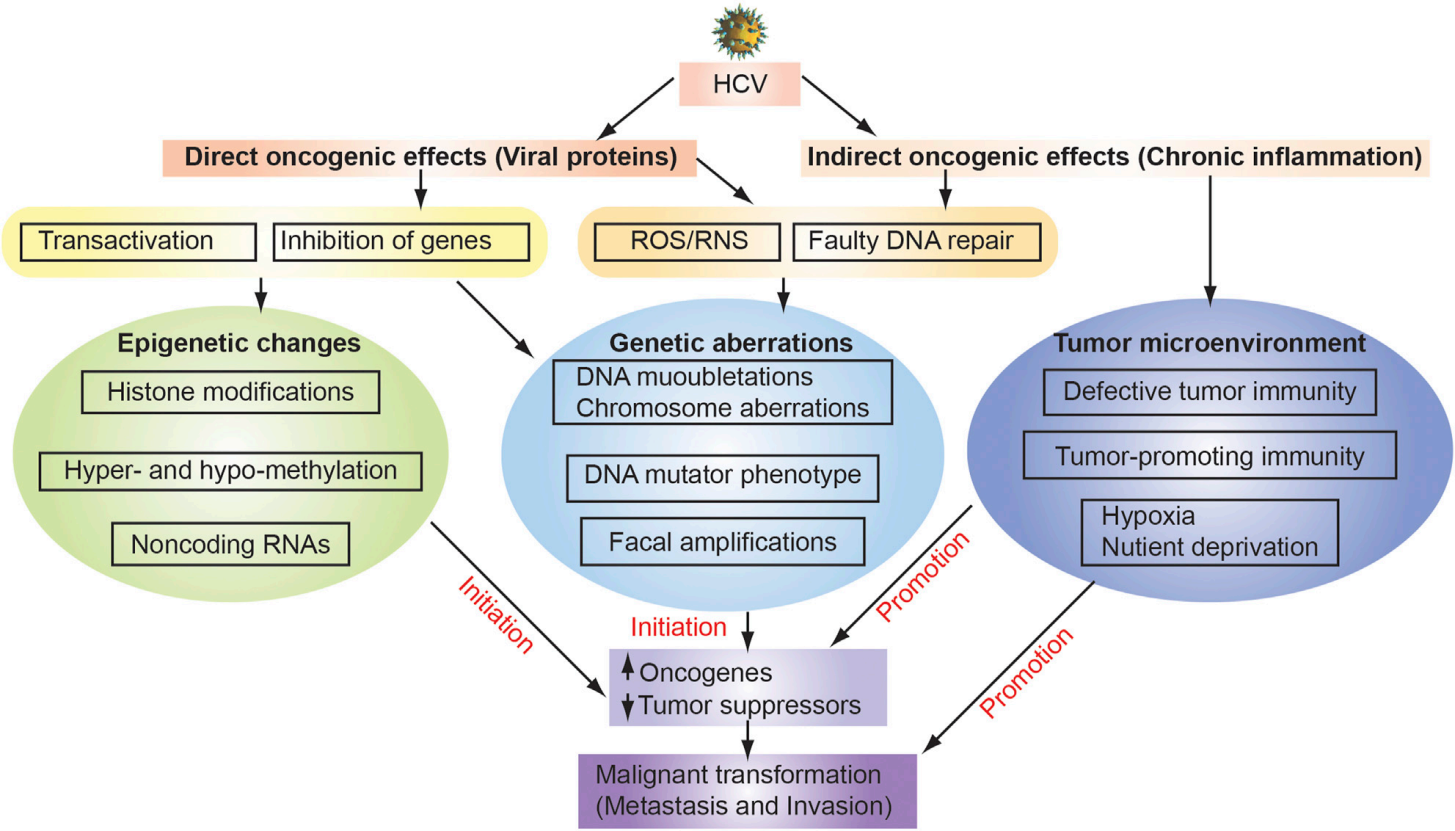
Mechanisms of HCV-associated hepatocarcinogenesis. HCV infection causes chronic inflammation in the liver as indirect oncogenic carcinogenic effects, leading to genetic alteration through ROS generation, elevated expression of DNA mutator phenotype, and dysfunction of DNA repair. As indirect oncogenic carcinogenic effects induce tumor microenvironment with defective tumor immunity, tumor-promoting immunity and hypocix and nutriet deprived environment. Environmental factors (e.g., Alcohol, obesity, and high fat diet) enhance sensitivity of livers to LPS. Direct effects of hepatitis virus, including the oncogenic effects of HBV genome integration and HBx protein expression, can also contribute to increased genomic instability. These multiple factors coordinately induce the accumulation of genetic and epigenetic alterations in liver tissue underlying chronic hepatitis or cirrhosis, leading to the development of HCC.

An HCV-induced oxidative environment may overwhelm cellular antioxidant and DNA-repair mchanisms, leading to accumulation of DSBs and chromosomal abnormalities in HCV-infected cells. As discussed above, HCV infection induces a mutator phenotype by causing DSBs (Machida et al., 2004a) through induction of iNOS mRNA and nitric oxide (NO) production by the viral core protein and the NS3 protein (Machida et al., 2004b). HCV non-structural protein NS3 blocks the cellular repair process. The ataxia-telangiectasia mutated kinase (ATM) is not only required for HCV replication (Ariumi et al., 2008) but also interacts with the HCV NS3-4A protein complex, resulting in impaired DNA damage responses and enhanced sensitivity to ionizing irradiation (Lai et al., 2008). The resulting increased rate of double-stranded DNA breaks is a possible direct viral causal role in tumorigenesis (Figure 3) (Lai et al., 2008).

DNA repair proteins prevent DNA mutations caused by oxidative damage, but are vulnerable to nitric oxide (NO)-induced oxidative damage. This is because of sulfhydryl, tyrosyl, and/or phenolic side chains in their active sites (Starke, Chen, Bapna, Lesnefsky, Mieyal; Jaiswal et al., 2000; Jaiswal et al., 2001). Suppression of DNA repair, coupled with the induction of DNA breaks by viral proteins, increases the mutation frequency and chromosome rearrangements in virus-infected cells. Indeed, HCV core proteins generate a chronic oxidative stress causing chromosomal and mitochondrial DNA instability (Hoshida et al., 2014).

The take-away message is that inhibition of DNA repair mechanism promotes mutator phenotype in HCV-associated HCC and these mutator phenotype should be reat targets for immune checkpoint inhibitor treatment (refer to Section 3). In cancer treatment, blocking poly (ADP-ribose) polymerase (PARP) prevents cancer cells from repairing their DNA damage, causing them to die (Buisson et al., 2010). Therefore PARP inhibitor treatment may be another strategy to reduce HCCs. The status of current related therapeutic strategies showed that PARP inhibitor inhibit many types of cancer development, possible for HCC as well. These genetic alterations induce self-rewnal ability of TICs.

## 3 Angiogenesis

Angiogenesis is elevated in highly vascularized tumors, including HCCs. The multi-tyrosine kinase inhibitor sorafenib (FDA-approved) is used in HCC patients to inhibit angiogenesis-inducing cytokine VEGF and the MAP Kinase, Raf/Mek/Erk pathways. Rapamycin is also used to inhibit the PI3K/Akt/mTOR pathway (Llovet and Bruix, 2008; Newell et al., 2009). Constitutive expression of Myc oncogene, platelet derived growth factor (PDGF), or VEGFA all lead to HCC development. Transgenic mouse studies demonstrated that tumor angiogenesis and recurrence is linked to MYC, PDGF and VEGFA pathways (Llovet and Bruix, 2008; Newell et al., 2009). Indeed, the angiogenesis biomarkers VEGF and Ang2 (angiogenin, ribonuclease A family, member 2) were independent predictors of advanced HCC patient survival (Llovet et al., 2012).

The take-away message is that angiogenesis is one of the most effective targets for HCC treatment since multi-kinase inhibitors Sorafenib and Rigorafenib are two FDA-approved chemotherapeutic drugs for HCC treatment after multinational, randomized, placebo-controlled, phase III Sorafenib HCC Assessment Randomized Protocol (SHARP) trial (Llovet et al., 2008). In HCC patients positive for anti-HCV antibody, sorafenib treatment improved median overall survival (OS), time to progression (TTP), disease control rate (DCR) (Llovet et al., 2008), indicating that these kinase signaling pathways maintain HCV-associated HCCs. New multikinase inhibitor Rigorafenib is used for Sorafenib-failure HCC patients.

## 4 Defective tumor immunity and HCC

Defective tumor immunity allows unrestricted HCC grow without tumor surveillace immune protection. HCV infection is a predisposing condition for HCC as viral clearance by the immune system since immune system cannot remove HCV-infected cells in almost all cases. Virus-specific CD8^+^ cytotoxic T lymphocytes (CTL) (Weiner et al., 1995; Cooper et al., 1999) clears HCV only in a minority (∼30%–40%) of cases (Hajarizadeh et al., 2013), leading to persistent lifelong infection with continuous immune-mediated hepatic inflammation.

Although mixed lymphocyte infiltration (T lymphocytes and NK cells) occurs in HCC tissues of patients (Rehermann, 2013), these immune cells are not cytoxic to cancer cells. Some of HCCs are tumors that do not have infiltration of immune cells, so called “cold” tumorsthat do not frequently respond to immune checkpoint inhibitor therapies. Indeed, HCV infection and/or viral proteins inhibit a variety of fuctions in many immune cell types, including CTL, CD4^+^ T, dendritic cells, macrophages and B cells (Bowen and Walker, 2005), indicating that HCV infection is associated with “cold” tumor phenotype.

Nonetheless infected hepatocytes do activate innate immunity by sensing HCV RNA motifs through RIG-I and TLR3. This leads to activation of the NF-κB pathway and generation of interferons and other pro-inflammatory cytokines. Furthermore the viral polymerase NS5B directly activates the inflammatory cascade through NF-κB in a MAVS and TBK-1 dependent manner, resulting in secretion of IL-6 and type I IFN (Yu et al., 2012).

Immune defects were targeted and translated into immunotherapies. Immunotherapy by the transplantation of donor bone-marrow stem cells kills tumor cells in the recipient (Dean et al., 2005). Thus, isolated TICs from a patient could be lethally irradiated and used to autologously ‘immunize’ the patient or used *ex vivo* activate donor immune cells against the patient’s TICs (Dean et al., 2005).

Immune checkpoint inhibitor anti-PD-1 therapy got FDA approval after landmark clinical trial (El-Khoueiry et al., 2017). As high genetic instability is good prognosis maker for immune checkpoint inhibitor, HCV-associated mutator phenotype (Machida et al., 2004a) can be great target for immune checkpoint inhibitor.

## 5 Immortalization

Human TERT cis-activation and telomerase enzymatic activity are associated with hepatocyte immortalization. HCV infection stimulates continuous growth and upregulates telomerase expression resulting in immortalization (Ray and Meyer, 2000). Furthermore, TERT promoter mutations are involved in TERT transactivation (Fujimoto et al., 2016). This immortalization process is prerequisite for step-wise carcinogenesis, which ultimately contributing to TIC formation.

Viral oncogenesis is characterized by two category, direct effects by viral proteins and indirect effects by inflammation (Figure 3). Direct oncogenic effects occur by expression of viral proteins, whereas indirect oncogenic effects occur by inflammation that is elicited by viral replication. HCV RNA is detected both in HCC and in surrounding non-tumor tissues (Alam et al., 2002; Sobesky et al., 2007). Both HCV structural and non-structural proteins are implicated in pro- and anti-apoptotic effects of hepatocytes, in eliciting inflammation and cancer-promoting signaling pathways, including WNT and sonic hedgehog pathways (McGivern and Lemon, 2011). Direct effects include viral protein-mediated transactivation or transcriptional suppression of genes, especially proto-oncogenes or tumor suppressor genes. Multifunctional HCV nucleocapsid core protein inhibits apoptosis, signal transduction, reactive oxygen species (ROS) formation, lipid metabolism, transcriptional activation, transformation and immune modulation. These additional hallmarks are observed in genesis of tumor-generating TICs when inoculated into immunocompromised mice. Therefore, a detailed and complete understanding of these mechanisms will contribute greatly to the development of new therapeutic strategies.

The take-away message is that HCV infection and/or viral proteins immortalize hepatocytes and/or cholangiocytes and/or liver progenitor cells through constitutive Telomerase activation. Telomere and/or other immrtalization siugnals can be targeted and translated into therapies. The status of current related therapeutic strategies include telomerase inhibitors and other kinase inhibitors.

## 6 Metabolic reprogramming

Compelling evidence identifies obesity/alcoholism and HCV as co-morbidity risk factors for HCC. The risk for HCC, as assessed by odds ratio, increases from 8 to 12 to 48–54, if HCV patients have concomitant obesity or alcoholism (Hassan et al., 2002; Yuan et al., 2004; Artinyan et al., 2010). Demographic data indicate increased HCC occurrence in african-americans, Hispanics/latinos, low income individuals and rural poor especially among alcoholics or obese patients in these populations. This synergism in HCC development is explained by gut microbiota changes to the TLR4-NANOG signaling pathways. As mentioned above the innate immune system induces the expression of pattern recognition molecular pattern Toll-like receptor 4 (TLR4) following HCV infection. This is observed in hepatocytes of transgenic mouse models, liver cell cultures and during natural infection. This induction of TLR4 sensitizes hepatocytes to endotoxemia induced by obesity and alcohol. Subsequent TLR4 signaling, induces the stem marker NANOG in the HCV nonstructural protein Ns5a transgenic (Tg) mice but not in wild type or Ns5a Tg mice deficient in TLR4. Only the combined effects of alcohol/obvesity and HCV infection lead to NANOG induction and liver tumors.

**FIGURE 4:**
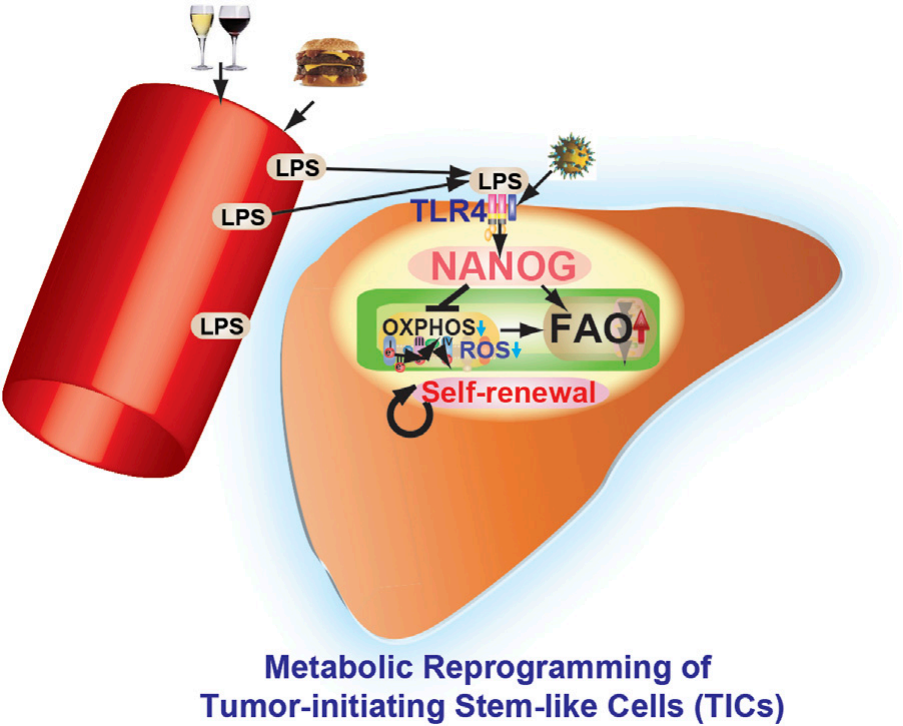
Pluripotency transcription factor NANOG contributes to cancer progression by mitochondrial reprogramming leading to the genesis of TICs. Environmental factors (alcohol and high-fat diet) and virus infection (i.e., hepatitis C virus) promote metabolic reprogramming and other characteristics of TICs. Obesity and alcoholism increase gut permeability leading to endotoxemia, which in turn activates Toll-like receptor 4 (TLR4) in the liver with induction of pluripotency transcription factor NANOG and an inflammatory response. This leads to subsequent development of obesity/alcohol-related liver cancer. NANOG ChIP-seq identified novel gene targets needed for oxidative phosphorylation (OXPHOS) and fatty acid oxidation (FAO). OXPHOS and fatty acid metabolism are identified as major pathways contributing to NANOG-mediated oncogenesis. NANOG-ChIP sequencing, gene profiling, proteomics, and metabolomics approaches were all combined to identify the altered pathway(s) in tumors. NANOG repressed OXPHOS and mitochondrial reactive oxygen species (ROS) in TICs. Restoration of OXPHOS and inhibition of FAO restored drug susceptibility of TICs. Identification of novel metabolic pathways provides potential drug targets for neutralizing the activity of highly malignant TICs found in cancer patients.

Obesity leads to persistent inflammation. This condition increases gut permeability allowing increased blood levels of endotoxins such as lipopolysaccharides (LPS), which in turn activate toll-like receptors (TLRs) (Hritz et al., 2008). TLR activation induces the production of cytokines and the inflammatory response, ultimately leading to liver injury and the development of obesity/alcohol-related liver disease (Figure 4). TLR4 activation in immune cells, however, does not induce stemness or pluripotency transcription factor, such as NANOG, without HCV infection or NS5A protein expression in transgenic mouse model, indicating that enhanced TLR4 expression induced by NS5A protein or other causal effects is required for excessive signaling in order to turn on NANOG. Furthermore, mature cells have an enhanced methylation on NANOG promoter while progenitor cells or embryonic stem cells have less methylation of NANOG promoter, indicating that hypomethylation status of NANOG promoter is also required for NANOG induction.

The TLR4-NANOG axis promotes metabolic reprogramming in hepatocytes *via* activation of fatty acid oxidation and inhibition of oxidative phosphorylation (OXPHOS) (Chen et al., 2016). Liver TICs are additionally sensitized to leptin and leptin exposure increases the expression and activity of an intrinsic pluripotency-associated transcriptional network comprised of STAT3, SOX2, OCT4, and NANOG. This axis is important but as previously mentioned, HCV infection is also required. This indicate that there are other predisposing factors for oncogenesis and tiC genesis beside TLR4 signaling activation.

Therefore, metabolic inhibitors and/or stemness inhibitors may prevent HCC self-renewal ability to kill HCC cells. Specific metabolic pathways may be novel therapeutic targets in order to selectively kill HCC cancer cells.

## 7 Excessive and prolonged inflammation

### 7.1 Indirect effects of chronic inflammation *via* a pro-carcinogenic environment

Chronic immune-mediated inflammation leads to repeated hepatocyte destruction and regeneration. As a consequence, these events lead to fibrogenic wound-healing responses which drive HCC or CC in chronic hepatitis C cases (Figures 1, 2). The prolonged liver inflammation coupled with the repeated liver regeneration process is conducive to multi-step hepatocarcinogenesis (Figure 3) (Simonetti et al., 1992).

Host pathogen-associated molecular pattern (PAMP) receptors sense double-stranded viral RNA replication intermediates to activate IFN Regulatory Factors IRF3/7 and NF-κB, leading to the induction of IFNs and related IFN-stimulated genes (ISGs). Persistent HCV infection induces ISG (Wieland et al., 2014) although HCV antagonizes RNA-sensor mechanisms for viral persistence (Li and Lemon, 2013). For negative feedback pathway, the HCV RNA replicase complex downregulates viral RNA synthesis, to maintain replication at low levels and minimizing oxidative damage (Yamane et al., 2014). HCV persistence induces hepatic oxidative DNA damage in chronic hepatitis C (Shimoda et al., 1994; Fujita et al., 2008).

### 7.2 Development of HCC in HCV patients with SVR and the role of TICs

Direct-acting antiviral agents might promote tumour occurrence in patients with cirrhosis, or recurrence in patients with presumed cure of hepatocellular carcinoma while DAA significantly reduces viral load. In view of the potential clinical implications, this controversy calls for a thorough and expeditious consideration of the hypothetical oncogenic activity of novel HCV drugs (Llovet and Villanueva, 2016).

Due to the “new” antiviral drugs available for HCV treatment, the incidence of HCC has changed. However, some patients who achieved sustained viral response develop HCC.

### 7.3 Toll-like receptor signaling

Mouse hepatocytes that express HCV-NS5A in liver upregulate the expression of Toll-like receptor 4 (TLR4) and develop liver tumors containing NANOG positive, tumor-initiating stem-like cells (TICs). The TLR signaling pathway is often upregulated in chronic liver diseases, especially since many different liver cell types express TLRs (Testro and Visvanathan, 2009). Hepatocytes express TLR1 through TLR9. Stellate cells express TLR2, 3, and 4. Bile duct epithelium expresses TLR2, 3, 4, and 5. Kupffer cells express TLR2, 3, and 4. Chronic alcohol consumption is associated with activation of TLR1, 2, and 6–9, which further increases the TNF-α response to LPS in mice (Testro and Visvanathan, 2009). Human monocytes exposed to ethanol for a week develop hypersensitivity to LPS through decreased IRAK-M expression, which activates mitogen-activated protein kinase (MAPK) and NF-κB following TLR4 signaling. This leads to activation of NF-κB, AP-1, and ERK (Mandrekar et al., 2009) and associated inflammatory response.

### 7.4 Nanog-positive TICs induced by virus and environmental factors (alcohol and obesity)

Nanog is one of the core transcription factors found in pluripotent embryonic stem cells (ESCs) (Martin, 1981). It is essential for maintaining self-renewal and pluripotency of both human and mouse embryonic stem cells (Loh et al., 2006; Wang et al., 2006; Rao and Orkin, 2006; Pan and Thomson, 2007). Overexpression of Nanog induces and maintains the pluripotency and self-renewing characteristics of ESCs under what normally would be differentiation-inducing culture conditions (Chambers et al., 2003). Recently, Nanog expression has been reported in human neoplasms, including germ cell tumors (Ezeh et al., 2005; Hart et al., 2005; Hoei-Hansen et al., 2005; Santagata et al., 2007), breast carcinomas (Ezeh et al., 2005), osteosarcoma (Gibbs et al., 2005), and HCC (Ma et al., 2008). Ectopic expression of Nanog induces an oncogenic potential in NIH3T3 (Zhang et al., 2005).

Nanog expression alone is not as effective as TLR4 activation in liver tumorigenesis, as shown by our cell transplantation experiments (Machida et al., 2009). TLR4 activation induces other tumor-driver genes which cooperatively work with Nanog to initiate liver oncogenesis. Thus, Nanog is essential for TLR4-dependent oncogenesis, but it alone is poorly oncogenic. This highlights the importance of alcohol and HCV NS5A synergism for liver tumor induction, especially in mice The importance of Nanog as a direct downstream gene of TLR4 in liver oncogenesis is summarized in Figure 4 (Feldman et al., 2012). Thus, pharmacologic inhibition of TLR4 signaling, including TLR4 antagonist (Eritoran or FP7) (Perrin-Cocon et al., 2017), may become a viable therapeutic strategy for HCV-associated liver tumors.

The take-away message is that TLR4-NANOG pathway is novel therapeutic targets.

The status of current related therapeutic strategies is under development.

## 8 Migration/invasion/metastasis

### 8.1 Epithelial-mesenchymal transition

Epithelial-mesenchymal transition promotes cell migration and invasion, ultimately leading to metastasis. This transition also occurs in development of HCC. As discussed earlier, the combined effect of TLR4-NANOG signaling promotes the development of TICs and tumorigenesis in transgenic mice expressing NS5A. These mice, when placed on a Western diet high in cholesterol and saturated fat (HCFD) activate the TLR4-NANOG axis in combination with the leptin receptor (OB-R)-pSTAT3 signaling pathways. The net resul is the occurrence of liver tumorigenesis through an exaggerated mesenchymal phenotype with prominent Twist1-expressing TICs (Uthaya Kumar et al., 2016).

Therefore, TWIST1 targetting therapy may prevent HCC cell spread into distal organs. Preventing metastasis spread would be effective strategy to keep HCC in primary organ site.

### 8.2 Epigenetic changes to sustain proliferation

Epigenetics is stable alterations in gene expression without genetic modifications in the sequence (Herceg, 2007). Epigenetic and genetic mechanisms silences cellular genes leading to transformation in human cancers, including HCC (Vaissiere et al., 2008). Contribution of different epigenetic factors, including genomic DNA methylation, histone modifications, and miRNA regulation, contribute to HCC dissemination, invasion, and metastasis. The reversal of deregulated epigenetic changes is emerging treatment of HCC (Nishida and Goel, 2011). High-throughput screening provides targeting inflammation-epigenome cross-talk in HCC to discover novel epigenetic targets (Herceg and Paliwal, 2011). Epigenetic Mechanism promotes the HBV/HCV-Related HCC tumorigenesis (Rongrui et al., 2014)

### 8.3 LncRNAs in HCV-associated HCC

Non-coding RNAs [LncRNA and microRNA (miRNA)] are dysregulated in HCV-induced liver carcinogenesis *via* regulation of gene expression. LncRNAs expression (LINC01419, AK021443, UCA1, and WRAP53) are increased in HCV-related HCCs compared to non-cancerous tissues (dysplasia) while lncRNA AF070632 is decreased in advanced HCC samples compared with early HCC. These lncRNAs are associated with Child-Pugh score. LINC01419 and AK021443 were mostly involved in cell cycle progression, whereas AF070632 regulates cofactor binding, oxidation-reduction and carboxylic acid catabolic process (Zhang et al., 2015). Two lncRNAs, including urothelial carcinoma associated-1 (UCA1) and WD repeat containing, antisense to TP53 (WRAP53) are upregulated in serum. UCA1 and WRAP53 (+) HCC patients had a decreased recurrence-free survival (RFS) and increased cumulative hazards. WRAP53 was an independent prognostic factor of RFS (Kamel et al., 2016). Some of these lncRNAs were dysregulated predominantly in one specific hepatitis virus-related HCC, including PCAT-29 in HBV-related HCC, aHIF and PAR5 in HCV-related HCC, and Y3 in HDV-related HCC. DBH-AS1, hDREH and hPVT1 were differentially expressed in HCC of different viral etiology (Zhang et al., 2016).

LncRNA and miRNAs have been associated with HCC (Wong et al., 2018). Due to this review being focused on TICs, more details about TICs and non-coding RNAs are described (such as (Huang et al., 2018; Machida, 2020; Rojas et al., 2022): More details about TICs and non-coding RNAs are included such as: (Huang et al., 2018; Machida, 2020; Rojas et al., 2022). In Table 1 HCV-associated HCC are involved in alterations of lncRNAs [(Hou and Bonkovsky, 2013; Lange et al., 2013; Zhang et al., 2015; Zhang et al., 2016; Fu et al., 2016; Shi et al., 2016; Hai et al., 2017; Wong et al., 2018; Sur et al., 2018; Toraih et al., 2018; Wang et al., 2018; Zhang et al., 2018; Cheng et al., 2019; Refai et al., 2019; Wang et al., 2019; Wu et al., 2019; Yang et al., 2019; Zhao et al., 2019; Zheng et al., 2019; Zhong et al., 2019; El-Khazragy et al., 2020; Ferrasi et al., 2020; Lorini et al., 2020; Machida, 2020; Mohyeldeen et al., 2020; Morishita et al., 2020; Oura et al., 2020; Unfried and Fortes, 2020; Jing et al., 2021; Sabry et al., 2021; Wong and Wong, 2021; Yao et al., 2021; Wang et al., 2022; Wei et al., 2022)]. Upregulated lncRNAs include NORAD (LINC00657), HCP5, lnc-HOTAIR (HOX antisense intergenic RNA), CASC11, HEIM, eosinophil granule ontogeny transcript (EGOT), lncRNA SEMA3B-AS1 [SEMA3B Antisense RNA 1 (Head To Head)], TPT-1S, LINC01189. In contrast, TPT1-AS1, LINC01152, aHIF and PWAR5 (PAR5) are downregulated.

**TABLE 1:** LncRNAs linked to HCV-associated HCC.

lncRNA	Classification	Size (kb)	Tissues	Expression	Function	Reference
BC017743	Unknown	2.3	Liver	Up	Tumor suppressor region	Zhang et al. (2016)
BC043430	Unknown	1.9	Liver	Up	Tumor suppressor region	Zhang et al. (2016)
LINC01152	Unknown	3.1	Liver	Down	Unknown	Zhang et al. (2016)
aHIF	Unknown	1.0	Liver	Down	Poor prognostic outcomes	Zhang et al. (2016)
PWAR5 (PAR5)	Unknown	∼3.6	Liver	Down	Poor prognostic outcomes	Zhang et al. (2016)
AF070632	Unknown	∼1.9	Liver	Down	LncRNA-protein interaction,supress angiogenesis,potential biomarker and therapeutic target	Zhang et al. (2015)
AK021443	Unknown	∼1.6	Liver	Up	Cell cycle regulation, Proliferation	Zhang et al. (2015)
LINC01419	Unknown	∼5.1	Liver	Up	Cell cycle regulation, Proliferation	Zhang et al. (2015)
UCA1	Unknown	∼7.3	Liver, bladder, gastric, ovary, esophagus	Up	LncRNA-miRNA interaction, Proliferation	Kamel et al. (2016)
WRAP53	Antisense	∼1.8	Liver	Up	Unknown, Biomarker	Kamel et al. (2016)
NORAD (LINC00657)	Unknown	5.3	Liver		Impairs Wee1 Expression, molecular decoy for PUMILIO proteins (PUM1/PUM2)	Sur et al. (2018)
HCP5	Unknown		Liver	Up	rs2244546 in HCP5 as a novel tagging SNP, a Hybrid HLA Class I Endogenous Retroviral Gene	Lange et al. (2013)
lnc-HOTAIR ((HOX antisense intergenic RNA)	Unknown	2.2	Liver	Up	master regulator of chromatin dynamics and cancer, Predict genotype 4 following direct-acting antivirals therapy	El-Khazragy et al. (2020)
CASC11	Unknown	0.52	Liver	Up	inhibiting miRNA-188-5p, ∼ 2.1 kb upstream of c-Myc	Cheng et al. (2019)
HAND2-AS1	Antisense		Liver, cervical cancer, osteosarcoma	Up	downregulating RUNX2 expression, represses cervical cancer progression by interaction with transcription factor E2F4, represses HIF1α-mediated energy metabolism	Kang et al. (2018); Jing et al. (2021)
PLAC2	Unknown		Liver	Down	Upregulates p53	Zheng et al. (2019)
SAMMSON	Unknown		Liver	Up	negatively regulates miR-9-3p, 30 kb downstream from MITF	Mohyeldeen et al. (2020)
HEIM	Unknown		Liver	Up	serum and exosomes as biomarker in the HCV-related HCC	Zhang et al. (2018)
eosinophil granule ontogeny transcript (EGOT)	Unknown		Liver, Head and neck squamous cell carcinomas (HNSCCs) a	Up	Increases the Expression of HMGA2 via Down-Regulating miR-33a-5p	Wu et al. (2019)
CASC2	Unknown		Liver	CASC2 Down	CASC2 was downregulated in HCC/HCV patients	Refai et al. (2019)
TUG1	Unknown		Liver	Up	TUG1 was overexpressed in relation to HCV and the control group, Tug1 lncRNA locus is essential for male fertility	Refai et al. (2019)
LINC01189	Unknown	1.4	Liver	Up	cell proliferation and chemoresistance through hsa-miR-155-5p	Yao et al. (2021)
lncRNA SEMA3B-AS1 (SEMA3B Antisense RNA 1 (Head To Head))	Antisense		Liver	Up	SEMA3B-AS1 in HCC tissues was inversely correlated with microRNA (miR)-718 and positively correlated with PTEN	Zhong et al. (2019)
MALAT1	Unknown	6.7	Liver	Up	Represent a putative non-invasive prognostic biomarker	Toraih et al. (2018)
TPT1-AS1	Antisense		Liver	Down	Suppresses HCC Cell Proliferation Downregulating CDK	Mohyeldeen et al. (2020)
NEAT1	Unknown	3.7 kb	Liver	Up	accurately differentiated between HCC patients and healthy controls, recruiting and binding to PRC2	Mohyeldeen et al. (2020)
TUG	Unknown	7.1 kb	Liver	Up	accurately differentiated between HCC patients and healthy controls, recruiting and binding to PRC2	Mohyeldeen et al. (2020)
Linc-p21	Unknown		Liver	Up	Unknown, Biomarker	Lim et al. (2019)
H19	Unknown		Liver	Up	Unknown, Biomarker	Lim et al. (2019)
LET	Unknown		Liver	Up	Unknown, Biomarker	Lim et al. (2019)
HULK	Unknown		Liver	Up	IGF2BP1 regulation, Biomarker	Lim et al. (2019)
HOTAIR	Unknown		Liver	Up	Guide of epigenetic repressors	Wong et al. (2018)
HOTTIP	Unknown		Liver	Up	Guide of epigenetic activators	Wong et al. (2018)

### 8.4 MicroRNAs

miRNA targets hundred mRNAs, miRs are diagnostic markers and therapeutic target for personalized therapy. miRNAs are differentially expressed in liver cancer and are related to different stages of liver carcinogenesis, supporting the diagnosis and prognosis tools of miRNAs in HCC patient. miRNAs promote or inhibits carcinogenesis via activation of oncogenes and/or suppression of tumor suppressors (Chen, 2005). HCV requires liver-specific miR-122 for replication (Jopling et al., 2005). Sequestering miR-122 in patients leads to a dose-dependent decrease in HCV viremia in phase 2a trial (Janssen et al., 2013). Mice lacking miR-122 have high tumor incidence (Tsai et al., 2012). The miR-122 abundance is reduced in human advanced fibrosis (Trebicka et al., 2013) and in therapresistant HCV patients (Sarasin-Filipowicz et al., 2009). Recruitment of miR-122 to the HCV genome depletes this important liver-specific miR-122. HCV sequesters anti-tumorigenic miR-122 to promote HCC development.

miR-21, miR-17, miR-222, miR-224, and miR-221 are increased in liver cancer (Borel et al., 2012) (Ladeiro et al., 2008) while miR-200, let-7, miR-29, miR-123, miR-122, miR-199a, and miR-199 b are decreased in HCCs (Hou et al., 2011; Huang and He, 2011; Anwar and Lehmann, 2015). miR-199a/b-3p prevents the p21-stimulated kinase 4/Raf/MEK/ERL pathway and suppresses HCC.

Down-regulation of miR-199a/b is associated with poor prognosis and low survival rate (Li et al., 2011). Increased miR-224 is associated with malignancy aggression, deteriorated liver function, and poor prognosis (Wang et al., 2008; Zhuang and Meng, 2015).

### 8.5 Histone modifications

Induction of HCV proteins or the infection of HCC cells with HCV cell culture (HCVcc) suppresses histone H4 methylation/acetylation and histone H2AX phosphorylation for HCC development, indicating that HCV-induced overexpression of PP2Ac are associated with HCC via deregulation of epigenetic histone modifications (Duong et al., 2010). HCV infection upregulates histone deacetylation (HDAC) activity through affecting hepcidin expression, a key suppressor of iron availability (Miura et al., 2008). The induced HCV oxidative stress leads to suppression of hepcidin expression by increased HDAC function. HCV increases histone deacetylation (HDAC) activity through negative regulator of iron availability (hepcidin expression) (Miura et al., 2008). Furthermore, antiviral agents IFN with epigenetic drugs (such as DNMT inhibitors or HDAC inhibitors) counteract epigenome changes with cytokines (Muller, 2006).

HCV caused epigenetic alteration mainly occurred on DNA repair-related genes. Induction of HCV proteins or the infection of HCC cells with HCVcc inhibits histone H4 methylation/acetylation and histone H2AX phosphorylation and inhibited DNA damage repair, indicating that HCV-induced overexpression of PP2Ac promotes hepatocarcinogenesis via dysregulation of epigenetic histone modifications (Duong et al., 2010).

### 8.6 Aberrant DNA methylation

HCV infection accelerates or inhibits the methylation process. DNA methyltransferases (DNMTs) methylates DNA. HCV upregulates DNA methyl transferases, which further block tumor suppressor genes leading to HCC (Tian et al., 2013). HCV core protein upregulates both mRNA and protein expression levels of DNMT1 and DNMT3b, which promotes DNA methylation in HCV-infected hepatocytes (Benegiamo et al., 2012). HCV core protein increases the mRNA and protein levels of DNMT1 and DNMT3b, leading to epigenetic alteration of HCV patients (Benegiamo et al., 2012). HCV tissues have seven hypermethylated markers (COX2, MINT1, CACNA1G, RASSF2, MINT2, Reprimo, and DCC) in comparison to both HBV and normal liver tissues (Nishida et al., 2008). Different HCV proteins NS5A (Kasprzak and Adamek, 2008) promotes hepatocarcinogenesis.

Epigenetic event Contribution in HCC development.

• DNA hypermethylation
  ○ CDH1: Cell adhesion and metastasis
  ○ RASSF1A: Cell cycle dysregulation
  ○ P21WAF1/CIP1: Cell cycle dysregulation
  ○ Gadd45: Response to genotoxic stress
  ○ MGMT: Dysfunction of DNA repair
  ○ APC: Cell cycle dysregulation/Dysfunction of DNA repair
• DNA hypomethylation (demethylation)
  ○ STAT1: Upregulation of JAK/STAT pro-tumorigenic signaling
  ○ COX-2: Inflammation
• Histone modification
  ○ PP2Ac: Inflammation

The adenomatous polyposis coli (APC) tumor suppressor gene encodes a large protein with multiple cellular functions and interactions, including signal transduction in the WNT-signaling pathway (Lee et al., 2006). The APC promoter is methylated in up to 81% of patients with viral hepatitis-induced HCC (Lee et al., 2003). A next generation sequencing of CpG methylation site demonstrates that APC was hypermethylated in HCC tissues to their corresponding non-tumorous tissues (Archer et al., 2010). In contrast, NOTCH4, EMR3, HDAC9, DCL1, HLA-DOA, HLA-DPA1, and ERN1 were hypomethylated in HCC (Archer et al., 2010).

The take-away message is that epigenetic regulation promotes envation, migration and metastatic characteristics thorugh aberrant expression of non-coding RNA, histone modification and DNA methylation. These aberrant epigenetic regulation can be targeted and translated into therapies. Indeed, miR-122 restoration strategy is being tested in clinical trials. The status of current related therapeutic strategies includes new miR and/or ncRNA targeted therapies.

## 9 Evading cell cycle arrest to sustain proliferation

### 9.1 Inactivation of tumor suppressor genes

Thel tumor suppressor p53 protein coordinates cell-cycle arrest, senescence, and apoptosis in response to DNA damage and cellular stresses (Bieging et al., 2014). Mutations in p53-DNAb-binding domains that disrupt DNA binding ability of p53 are associated with many human cancers, including HCC (Hussain et al., 2007; Guichard et al., 2012). HCV proteins target tumor suppressor genes and proto-oncogenes. For example, three HCV proteins, including core, NS3, and NS5A, interact with tumor suppressor p53 when overexpressed in cell culture (McGivern and Lemon, 2011). There is conflicting data on whether core interaction with p53 results in activation or inhibition of p53 target genes, but thismay reflect differences in the level of core expression (high level core expression is needed) (Kao et al., 2004). The NS3-p53 interaction blocks apoptosis *in vitro* (Deng et al., 2006) and NS5A interaction with p53 results in p53 redistribution to the peri-nuclear membrane (Majumder et al., 2001). The impact of HCV proteins on p53 activity and interactions between HCV proteins and p53 are controversial since the cell-lines that are most permissive for HCV (Huh-7 hepatoma cells and their derivatives: Huh7.5 and Huh7.5.1) express a mutated, inactive form of p53 (Bressac et al., 1990; Hsu et al., 1993). Additionally, the retinoblastoma tumor suppressor protein (Rb) interacts with the HCV NS5B protein, leading to its poly-ubiquitination and degradation to promote S phase entry (Munakata et al., 2005). The take-away message is that HCV infection and/or viral proteins inactivates tumor suressor pathways. Therefore, restoration of p53 function, by use of Adenovirus expressing functional p53, may restore tumor suppressor function and selectively kill HCC cells.

### 9.2 Constitutive activation of cell cycle

HCV infection modulates several genes, including SOCS-1 Suppressor of cytokine signaling (SOCS) 1 (negative regulator of the JAK/STAT pathway). Expression of SOCS1 leads to reduced JAK /STAT phosphorylation, reduced STAT dimerization and imports to the nucleus and reduced transcription of target genes (Chim et al., 2004). JAK/STAT pathway is pro-tumor signaling (Calvisi et al., 2006). The take-away message is that HCV infection and/or viral proteins constitutively activate proto-oncogenes and/or inhibits tumor suppressors. Therefore, STAT3 is targeted and translated into STAT3 inhibitor therapies.

## 10 Resistance to cell death of TICs

Stem cells have three major characteristics: self-renewal, asymmetric and multiple cell division (clonality), and plasticity. Hepatic small oval progenitor cells around the peripheral branches of the bile ducts, the canals of Hering, differentiates into biliary epithelial cells and hepatocytes (Roskams et al., 2004). These oval liver progenitor cells share molecular markers with adult hepatocytes [albumin, cytokeratin 7 (CK7), CK19, oval cell markers (OV-6, A6, and OV-1), chromogranin-A, NCAM (neural cell adhesion molecule)] and fetal hepatocytes (α-fetoprotein) (Roskams et al., 2004; Roskams, 2006). They are also positive for more common stem cell markers such as CD34^+^, Thy-1+ (Burke et al., 2007). A CD117+/CD133+ hepatic precursors are detected in regenerating liver tissue (Craig et al., 2004) while a CD45–/CD90+ tumor subpopulation are detected in HCC (Yang et al., 2008). The CD90^+^ cells are not present in the normal liver and, when injected into immunodeficient mice, create tumors repeatedly. In human HCC and HCC cell lines, specifically CD133(+) cells, not CD133(−)cells, had the ability to self-renew, create differentiated progenies, and form tumors (Ma et al., 2007).

Forty percent of HCC have clonality, and thus are considered to originate from progenitor/stem cells (Alison, 2005; Roskams, 2006; Zender et al., 2006; Tang et al., 2008). TICs express stemness genes, including CD133 (Prominin in mice), Wnt/β-catenin, Nanog (Feldman et al., 2012), Notch, Hedgehog/SMO, Bmi, Oct3/4 (Beachy et al., 2004; Chambers and Smith, 2004; Valk-Lingbeek et al., 2004), NOTCH, BMI, OCT3/4, CD44 (cell adhesion molecule) and CD34. The CD133 subpopulations displayed similar expression for CD29 (integrin β1), CD49f (integrin α6), CD90, and CD117 (c-kit: gastrointestinal stroma tumor), indicating these makers are still not definitive TIC markers (Ma et al., 2007). CD133+/CD49f + HCC TICs confer resistance to chemotherapy, which hampers efficacy of therapy in HCC (Rountree et al., 2008). Thus, HCV infection is associated with TICs and HCC development.

Dysregulated signaling and gene expression promotes the plasticity of TICs to resist current FDA-approved therapies, such as sorafenib or regorafenib or anti-PD1 immune checkpoint inhibitor treatment. TICs of HCC are observed to have several elevated oncogenic and anti-apoptotic signaling pathways such as PI3K/AKT (Ma et al., 2008), signal transducer and activator of transcription 3 (STAT3) (Wurmbach et al., 2007; Yeoh et al., 2007), Notch (Dando et al., 2005), hedgehog (Sicklick et al., 2006a; Sicklick et al., 2006b) and transforming growth factor-beta (TGF-β) (Kitisin et al., 2007; Nguyen et al., 2007).

Normal stem cells and TICs express high levels of ATP-binding cassette (ABC) transporters, such as *ABCB1*, and the half-transporter *ABCG2* identified in mitoxantrone-resistant cells (Shepard et al., 2003; Schneiderman et al., 2010). Cancer cells in culture become resistant to cytotoxic anticancer drugs through multiple pathways, such as increased active efflux at the plasma membrane (MDR1, MRP family members, and MXR), reduced drug uptake, expression of one or more energy-dependent transporters that specifically detect and eject intracellular anticancer drugs, insensitivity to drug-induced apoptosis and induction of drug-detoxifying mechanisms (Gottesman, 2002). The drug-transporting property of TICs conferred by ABC transporters is the basis for the observed ‘side-population’ phenotype that is identified by exclusion of the fluorescent dye Hoechst 33,342. Therefore, tumors may have an intrinsic population of drug-resistant pluripotent cells that survive chemotherapy and thus repopulate the tumor (Gottesman, 2002). The take-away message is that drug-transporting property of TICs are rationale target to suppress drug-resisstance phenotype of TICs. Therefore, ABCG2 inhibitors (GF120918 and tariquidar) will inhibit both ABCG2 and ABCB1 to overcome drug resistance by inhibition of the Hedgehog-Patched receptor signaling protein, Smoothened.

### 10.1 Compensatory regeneration and proliferation

Chronic liver inflammation causes liver injury with compensatory hepatocellular proliferation which indirectly promotes HCC. Regenerative pathways promote dedifferentiation and proliferation to replace damaged tissue in inflammatory liver disease and hepatocellular necrosis associated with chronic hepatitis C infection.

For example, the Wnt/β-catenin pathway is directly altered by HCV. Initially, NS5A promotes β-catenin stabilization through inactivation of GSK3-β, which normally promotes β-catenin degradation (Street et al., 2005; Park et al., 2009). Addiotional study shows that NS5A directly interacts with β-catenin and stabilizes β-catenin (Milward et al., 2010). Similarly GSK3-β is also a target of hepatitis B virus X-protein which results in stabilization of β-catenin. HCV interacts with the TGF-β pathway leading to cytostatic response or fibrogenic responses. HCV NS5A blocks TGF-β signaling through direct interaction with its receptor, TGF-β receptor I (TβR-I) (Choi and Hwang, 2006). Antagonism of TGF-β signalingpromotes liver damage, fibrosis and cancer. Furthemore, HCV core variant isolated from HCC, but not from surrounding liver tissue blocks TGF-β signaling through interactions with SMAD3 (Pavio et al., 2005).

Cells within such a precancerous field contain mutations that predispose progression to a cancerous phenotype. What is the impact of chronic hepatocyte turnover on tumorigenesis? Perhaps apoptosis of infected hepatocytes,either by immune- or virus-mediated mechanisms with compensatory hepatocellular proliferation, promotes carcinogenesis during decades of chronic HCV infection.

The association of liver injury with HCV infection is depicted in Figure 4. Core protein activates cellular oncoproteins and NF-*κ*B cell signaling pathways and causes p53 and pRb inactivation to initiate genomic instability and uncontrollable cellular proliferations (Smirnova et al., 2006). HCV nonstructural proteins NS5A and NS3 alter host expression and promote liver cell proliferation, leading to HCC development (Jeong et al., 2012). NS5A suppresses immune responses, inactivates tumor suppressors, inhibits apoptosis, and disrupts liver homeostas, thus leading a to a primary liver.

The take-away message is that HCV promotes HCC growth by compensatory regeneration and proliferation. Compensatory regeneration and proliferation were targeted and translated into therapies. the status of current related therapeutic strategies showed sorafenib and regorafenib inhibits compensatory regeneration and proliferation.

### 10.2 Concluding Remarks

**FIGURE 5:**
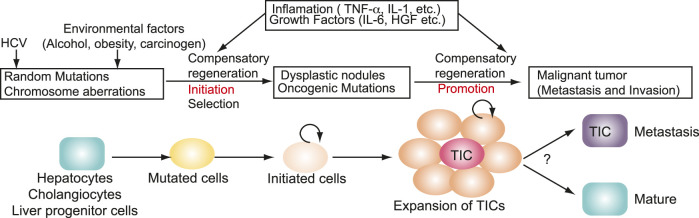
Genesis of TICs in HCV carcinogenesis. Environmental factors (alcoholism, obesity and carcinogen exposure) and HCV infection are invoved in initiation and promotion. Transformed cells are further altered and progressed to TICs. Expansion of TICs requires self-renewal property. TICs acuired tumor-initiation property.

The TIC population is a prime origin of cancer recurrence in drug-treated patients with HCC. TICs are generated in HCV carcinogenesis (Figure 5). TICs acuired tumor-initiation property. The molecular pathways of HCV-induced carcinogenesis may involve indirect, non-virological factors such as the induction of chronic liver inflammation and regeneration that lead to the emergence of mutated cells with high proliferation rates. In addition, HCV infection may also involve viral gene products that stimulate the production of ROS with the expression of error-prone DNA polymerases. These diverse pathways highlight the complicated interplays between the virus and its host in HCV associated carcinogenesis. Given the latter pathway, potential use of ROS and or iNOS inhibitors may be useful for treating HCC patients with HCV co-morbidity. Transformed cells are further altered and progressed to TICs (Figure 5). Expansion of TICs requires self-renewal property (Figure 5). Environmental factors (alcoholism, obesity and carcinogen exposure) and HCV infection are invoved in initiation and promotion (Figure 5). Chronic HCV infection results in a high frequency of HCC that displays non-metastatic and multicentric characteristics.

Additional therapies are desperately needed since current treatments (sorafenib, regorafenib or anti-PD1 immune checkpoint inhibitors) have limitationsbecause eventual treatment failures leads to cancer metastasis. Future investigative projects need to address the specific treatment needs of patients with HCC. Current FDA-approved immunotherapy, such as anti-PD-1 or anti-CTLA4 therapy has a limited efficacy for a small fraction of HCC patients (10%–25% range undergoing monotherapy). The remaining HCC patients did not respond to this monotherapy, indicating that an immune-checkpoint inhibitor approach has a limited efficacy and other immune mechanisms may be needed to have synergism with tumor-killing cells, such as antigen presenting cells, including dendritic cells and B cells. However inclusion of immune checkpoint inhibitors with combination therapy may break immune tolerance and improve the therapeutic efficacy of this approach.

Simply extending patient life spans by several months is not suffiecient. The goal should be to expand the completely cured patient population. This is the biggest challenge of researchers to transform a “previously “incurable” malignancy into a curable illness. Discovery of new immune checkpoint inhibitors and incorporation into combination therapies may be a new therapeutic avenue to drastically improve HCC treatment. HCC should not be a death sentence and may become a curable malignancy providing foundational research findings can be translated into game-changing innovative treatments (Refer to the outstanding questions box).

Finally, personalized medicine approaches will stratify the HCC patient population into distinct subpopulations that may be responsive to HCC-type specific treatments. As presented in this review, there are several avenues of liver morbidities leading to HCC. Thus, treatment options may likely reflect the original triggering event leading to HCC.

## Appendix A: Text box

### Appendix B: Genetically modified models (GMM) by insertion of hepatitis C viral genes

Genetically modified mouse models identify molecular and histological stages during the process of multistep hepatocarcinogenesis. Because of the large number of transgenic mice, only a small selection of representative models for HCC research are discussed in this review (Table A1).

**TABLE A1:** Summary of transgenic models.

	Promotor	Time to develop tumors	% Of mice with HCC	References
am				
HCV	Albumin	90–100 weeks	15	Lerat et al. (2002)
HCV core	HBV	80–105 weeks	32	Moriya et al. (1998); Tanaka et al. (2008)
HCV core	Albumin	+DEN: 32 weeks	100	Kamegaya et al. (2005)
HCV Core-NS2	CMV	50 weeks	25–30	Wakita et al. (1998)
E1-E2	HBV	60 weeks	23	Naas et al. (2005)

#### Appendix C: Trends Box 1. Epigenetic changes

Epigenetics is stable alterations in gene expression without genetic modifications in the DNA sequence (Herceg, 2007). Epigenetic and genetic mechanisms silences cellular genes leading to transformation in human cancers, including HCC (Vaissiere et al., 2008). Contribution of different epigenetic factors, including genomic DNA methylation, histone modifications, and miRNA regulation, contribute to HCC dissemination, invasion, and metastasis. The reversal of deregulated epigenetic changes is emerging treatment of HCC (Nishida and Goel, 2011). High-throughput screening provides targeting inflammation-epigenome cross-talk in HCC to discover novel epigenetic targets (Herceg and Paliwal, 2011). Epigenetic Mechanism promotes the HBV/HCV-Related HCC tumorigenesis (Rongrui et al., 2014). Several LncRNAs regulate cancer biology, including MALAT and etc. LncRNAs have pleiotropic effects on miRNAs, mRNAs, and proteins and alter miRNA and mRNA expression and stability, affect protein expression, degradation, structure, or interactions with transcriptional regulators. Therefore, novel lncRNAs serve as biomarkers to achieve precision therapy for HCC (Lim et al., 2019). The function of the 98% of non-coding sequences in the human genome is elusive (Wong et al., 2018).

##### Appendix D: Trends Box 1.1. MicroRNAs

Antiviral agents INF with epigenetic drugs (such as DNMT inhibitors or HDAC inhibitors) counteract epigenome changeseract epigenome changes with cytokines (Muller, 2006). Induction of HCV proteins or the infection of HCC cells with HCV cell culture (HCVcc) suppresses histone H4 methylation/acetylation and histone H2AX phosphorylation for HCC development, indicating that HCV-induced overexpression of PP2Ac are associated with HCC via deregulation of epigenetic histone modifications (Duong et al., 2010). HCV infection upregulates histone deacetylation (HDAC) activity through affecting hepcidin expression, a key suppressor of iron availability (Miura et al., 2008). The induced HCV oxidative stress leads to suppression of hepcidin expression by increased HDAC function. Non-coding RNAs [microRNA (miRNA)] are dysregulated in HCV-induced liver carcinogenesis via regulation of gene expression. miRNA targets hundred mRNAs, miRs are diagnostic markers and therapeutic target for personalized therapy. miRNAs promote or inhibits carcinogenesis via activation of oncogenes and/or suppression of tumor suppressors (Chen, 2005). miRNAs are differentially expressed in liver cancer and are related to different stages of liver carcinogenesis, supporting the diagnosis and prognosis tools of miRNAs in HCC patient. HCV requires liver-specific miR-122 for replication (Jopling et al., 2005). Sequestering miR-122 in patients leads to a dose-dependent decrease in HCV viremia in phase 2a trial (Janssen et al., 2013). Mice lacking miR-122 have high tumor incidence (Tsai et al., 2012). The miR-122 abundance is reduced in human advanced fibrosis (Trebicka et al., 2013) and in therapresistant HCV patients (Sarasin-Filipowicz et al., 2009). Recruitment of miR-122 to the HCV genome depletes this important liver-specific miR-122. HCV sequesters anti-tumorigenic miR-122 to promote HCC development. miR-21, miR-17, miR-222, miR-224, and miR-221 are increased in liver cancer (Borel et al., 2012) (Ladeiro et al., 2008) while miR-200, let-7, miR-29, miR-123, miR-122, miR-199a and miR-199 b are decreased in HCCs (Hou et al., 2011; Huang and He, 2011; Anwar and Lehmann, 2015). miR-199a/b-3p prevents the p21-stimulated kinase 4/Raf/MEK/ERL pathway and suppresses HCC. Downregulation of miR-199a/b is associated with poor prognosis and low survival rate (Li et al., 2011). Increased miR-224 is associated with malignancy aggression, deteriorated liver function, and poor prognosis (Wang et al., 2008; Zhuang and Meng, 2015).

#### Appendix D: Trends Box 1.3. Histone modifications

HCV caused epigenetic alteration mainly occurred on DNA repair-related genes. Induction of HCV proteins or the infection of HCC cells with HCVcc inhibits histone H4 methylation/acetylation and histone H2AX phosphorylation and inhibited DNA damage repair, indicating that HCV-induced overexpression of PP2Ac promotes hepatocarcinogenesis via dysregulation of epigenetic histone modifications (Duong et al., 2010). HCV increases histone deacetylation (HDAC) activity through negative regulator of iron availability (hepcidin expression) (Miura et al., 2008).

##### Appendix D: Trends Box 1.4. Aberrant DNA methylation

HCV infection accelerates or inhibits the methylation process. DNA methyltransferases (DNMTs) methylates DNA. HCV upregulates DNA methyl transferases, which further block tumor suppressor genes leading to HCC (Tian et al., 2013). HCV core protein upregulates both mRNA and protein expression levels of DNMT1 and DNMT3b, which promotes DNA methylation in HCV-infected hepatocytes (Benegiamo et al., 2012). HCV core protein increases the mRNA and protein levels of DNMT1 and DNMT3b, leading to epigenetic alteration of HCV patients (Benegiamo et al., 2012). HCV tissues have seven hypermethylated markers (COX2, MINT1, CACNA1G, RASSF2, MINT2, Reprimo, and DCC) in comparison to both HBV and normal liver tissues (Nishida et al., 2008). Different HCV proteins NS5A (Kasprzak and Adamek, 2008) promotes hepatocarcinogenesis. Epigenetic event Contribution in HCC development.

• DNA hypermethylation
  ○ CDH1: Cell adhesion and metastasis
  ○ RASSF1A: Cell cycle dysregulation
  ○ P21WAF1/CIP1: Cell cycle dysregulation
  ○ Gadd45: Response to genotoxic stress
  ○ MGMT: Dysfunction of DNA repair
  ○ APC: Cell cycle dysregulation/Dysfunction of DNA repair
• DNA hypomethylation (demethylation)
  ○ STAT1: Upregulation of JAK/STAT pro-tumorigenic signaling
  ○ COX-2: Inflammation
• Histone modification
  ○ PP2Ac: Inflammation

HCV infection modulates several genes, including SOCS-1 Suppressor of cytokine signaling (SOCS) 1 (negative regulator of the JAK/STAT pathway). Expression of SOCS1 leads to reduced JAK /STAT phosphorylation, reduced STAT dimerization and imports to the nucleus and reduced transcription of target genes (Chim et al., 2004). JAK/STAT pathway is pro-tumor signaling (Calvisi et al., 2006).

The adenomatous polyposis coli (APC) tumor suppressor gene encodes a large protein with multiple cellular functions and interactions, including signal transduction in the WNT-signaling pathway (Lee et al., 2006). The APC promoter is methylated in up to 81% of patients with viral hepatitis-induced HCC (Lee et al., 2003). A next generation sequencing of CpG methylation site demonstrates that APC was hypermethylated in HCC tissues to their corresponding non-tumorous tissues (Archer et al., 2010). In contrast, NOTCH4, EMR3, HDAC9, DCL1, HLA-DOA, HLA-DPA1, and ERN1 were hypomethylated in HCC (Archer et al., 2010).
